# Over‐the‐catheter endoscope replacement for stenting in patients with inaccessible malignant colonic obstruction with coexisting peritoneal carcinomatosis

**DOI:** 10.1111/den.14385

**Published:** 2022-08-09

**Authors:** Yoichiro Iboshi, Yorinobu Sumida, Eikichi Ihara, Hiroyuki Fujii, Naohiko Harada, Makoto Nakamuta, Yoshihiro Ogawa

**Affiliations:** ^1^ Department of Gastroenterology Clinical Research Institute National Hospital Organization Kyushu Medical Center Fukuoka Japan; ^2^ Department of Medicine and Bioregulatory Science Graduate School of Medical Sciences, Kyushu University Fukuoka Japan; ^3^ Department of Gastroenterology and Hepatology National Hospital Organization Fukuokahigashi Medical Center Fukuoka Japan

**Keywords:** colonic neoplasm, colonoscopy, intestinal obstruction, peritoneal carcinomatosis, self‐expandable metallic stent

## Abstract

Although a large‐caliber endoscope (LCE) is indispensable for through‐the‐scope placement of a self‐expandable metallic stent (SEMS) in patients with malignant colonic obstruction (MCO), inaccessibility of the target obstructing lesion (TOL) by the endoscope is a significant cause of unsuccessful procedures. We herein present a novel salvage procedure when the TOL is not directly accessible by an LCE in conditions such as coexisting peritoneal carcinomatosis involving the colon. The salvage procedure, termed over‐the‐catheter endoscope replacement (OCER), starts with an ultraslim endoscope suitable for deep insertion beyond a tortuous colon for traversing a guidewire through the TOL. The ultraslim endoscope is then withdrawn and replaced by an LCE through the following steps. An endoscopic retrograde cholangiopancreatography catheter is preloaded in the LCE, the catheter alone is passed over the guidewire already traversed through the TOL, and the LCE is navigated over the catheter as far as possible toward the TOL to deliver the SEMS delivery system in a standard through‐the‐scope manner or further in an over‐the‐wire manner even if LCE insertion is incomplete. Among the 165 patients with MCO who underwent stenting during our study period, OCER led to successful procedures in all nine patients whose TOLs were initially inaccessible because of colon‐involving peritoneal carcinomatosis. By utilizing the functions of distinctive endoscopes in a unique and complementary way, OCER can be a practical salvage option for challenging cases of MCO that are highly prone to unsuccessful palliation by conventional SEMS placement.

## INTRODUCTION

Colonic self‐expandable metallic stent (SEMS) placement is an alternative to surgical intervention in the management of malignant colonic obstruction (MCO), especially in palliative settings.^1^, ^2^ The through‐the‐scope (TTS) procedure and instrumental advancements have made each step of SEMS placement precise and stable with a high success rate, and the TTS procedure has thus become the standard procedure for colonic SEMS placement.^3^, ^4^, ^5^, ^6^

The initial step of the conventional TTS procedure is to access the target obstructing lesion (TOL) by inserting a large‐caliber endoscope (LCE) with a large working channel that accommodates the SEMS delivery system.^3^, ^4^, ^5^, ^6^ However, this step is often challenging in patients with MCO with coexisting peritoneal carcinomatosis because it causes narrowing, adhesion, and tortuous changes of the involved colon.^6^, ^7^ Failure to fulfill this minimum requirement leads to failed stent placement that warrants urgent surgery, which is associated with increased morbidity and mortality.^8^ Thus, a technical improvement to solve this problem is needed.

We herein present a salvage procedure termed over‐the‐catheter endoscope replacement (OCER) for failed insertion of the LCE to the TOL during conventional SEMS placement in patients with MCO. OCER allows the choice of an ultraslim endoscope^9^, ^10^, ^11^ for initial access to the TOL and its replacement by an LCE with the help of a commonly used endoscopic retrograde cholangiopancreatography (ERCP) catheter combined with a guidewire. This novel technique provides an opportunity to complete the SEMS placement when patients with MCO need decompression but their clinical condition, such as peritoneal carcinomatosis, technically prevents the conventional SEMS placement procedure.

## PROCEDURE

### Patients

**Figure 1:**
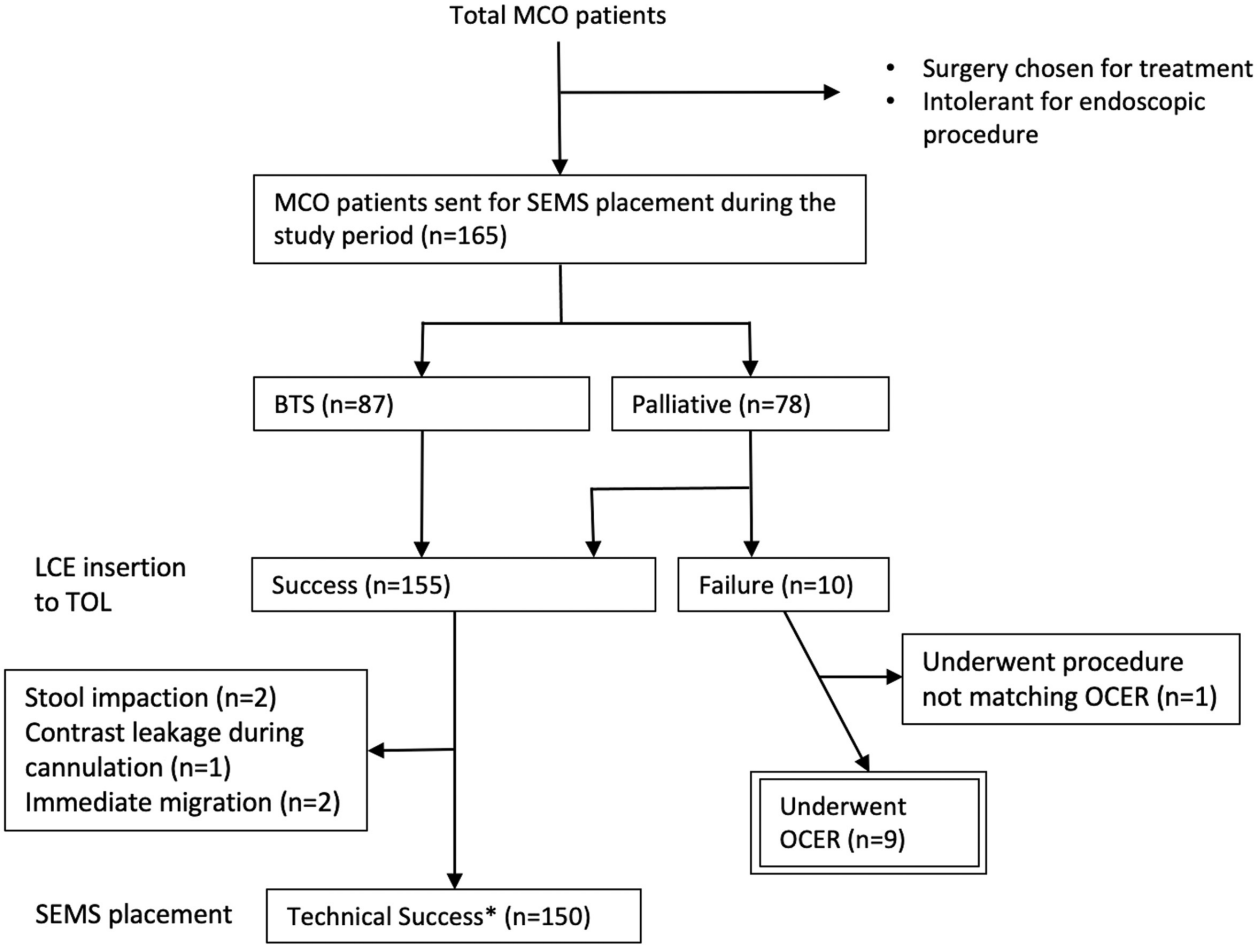
Flowchart of 165 patients with malignant colonic obstruction (MCO) who underwent standard self‐expandable metallic stent (SEMS) placement and the salvage procedure (over‐the‐catheter endoscope replacement, OCER) during the study period. The patients underwent SEMS placement unless surgery was preferred or the patients were unable to tolerate the endoscopic procedure. Among the 165 patients who underwent standard SEMS placement, the success rate of large‐caliber endoscope (LCE) insertion to the target obstructing lesion (TOL) was 100% (87/87) in the bridge to surgery (BTS) group and 82% (68/78) in the palliative group. Out of 10 patients in the palliative group in whom an LCE could not be inserted to the TOL, nine underwent the salvage procedure (OCER). The other patient underwent a similar procedure that did not meet the OCER definition (not using an ultraslim endoscope). When limited to the 155 patients in whom an LCE could be inserted to the TOL, SEMS placement was technically successful in 96.8% of cases (150/155); the five failed cases were related to inadequate early drainage by stool impaction (*n* = 2) in the BTS group, immediate migration (*n* = 2), and contrast leakage (perforation or fistula formation) (*n* = 1) in the palliative group. *Defined as placement of a stent or stents over the entire stricture with passage of stool or injected contrast media from the proximal to distal side of the stricture.

**Figure 2:**
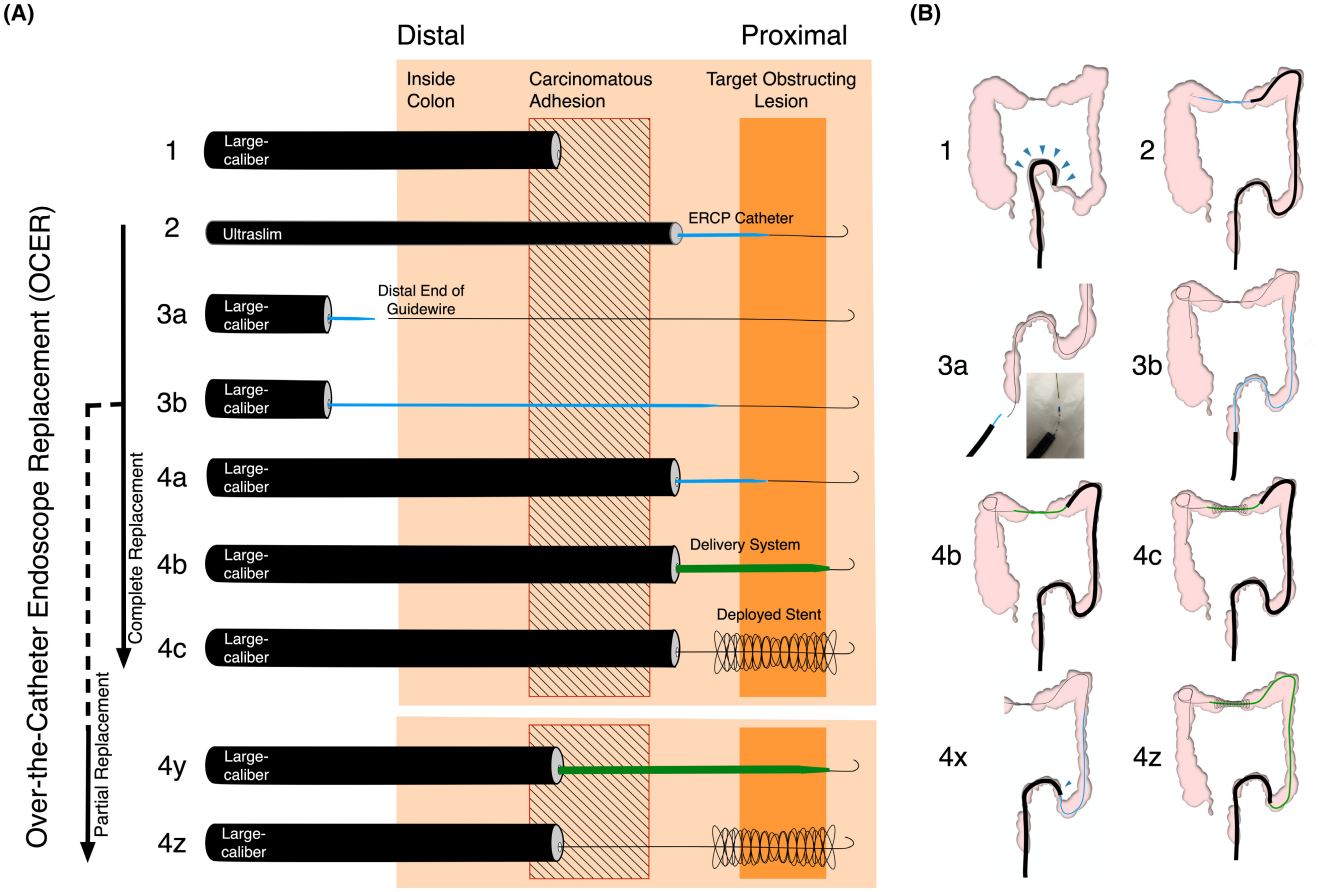
Procedural steps of over‐the‐catheter endoscope replacement (OCER) for self‐expandable metallic stent (SEMS) placement in a patient with inaccessible malignant colonic obstruction with coexisting peritoneal carcinomatosis. (A) Each step of OCER for SEMS placement is shown. The target obstructing lesion (TOL) is shown as an orange area, and carcinomatous adhesion associated with peritoneal carcinomatosis is shown as a hatched area. (B) Schemata of each step of OCER in a representative case of an obstructing lesion at the transverse colon and a carcinomatous adhesion in the sigmoid colon. The labeled numbers in (B) correspond to those in (A). When a large‐caliber endoscope (LCE) cannot pass through the colonic segment affected by carcinomatous adhesion (A‐1, arrowheads in B‐1), an ultraslim colonoscope is inserted to the TOL, followed by an endoscopic retrograde cholangiopancreatography (ERCP) catheter combined with a guidewire passed through the stricture (A‐2, B‐2). Only the guidewire is then left in the colon. Outside the body, the ERCP catheter is preloaded in the LCE (A‐3a, B‐3a). The catheter is passed over the guidewire left in the colon until it reaches the TOL (A‐3b, B‐3b). The LCE is advanced over the catheter (A‐4a). The catheter is removed and the SEMS delivery system is inserted in a conventional through‐the‐scope manner (A‐4b, B‐4b) for deployment (A‐4c, B‐4c). If the LCE cannot be inserted up to the stricture (partial replacement, B‐4x), the delivery system by itself is advanced for the remaining distance to the TOL over the guidewire under fluoroscopy (A‐4y) to complete SEMS placement in a remotely controlled manner (A‐4z, B‐4z). [Colour figure can be viewed at wileyonlinelibrary.com]

From January 2014 to August 2020, a total of 165 patients with MCO diagnosed by their symptoms, medical histories, and computed tomography findings consecutively underwent colonic SEMS placement unless surgical intervention was favored (Fig. 1). The clinical characteristics, including the cause and nature of the obstruction, of the 165 patients are shown in Table S1. Eighty‐seven of these patients underwent SEMS placement in preoperative settings as a bridge to surgery for colorectal cancer, and the other 78 patients underwent SEMS placement in palliative settings for treatment of various types of metastatic malignancies. Out of 10 patients who underwent unsuccessful insertion of an LCE to the TOL in an attempt to perform standard endoscopic stenting, nine underwent a salvage procedure using OCER (Fig. 1) as described later (Fig. 2). Information regarding the clinical characteristics and details of the stenting procedure, technical outcome, adverse events, and symptom changes (ColoRectal Obstruction Scoring System [CROSS] score^12^, ^13^) was collected from the medical records and the prospectively formatted endoscopy database (Table S1). Each patient gave informed consent to undergo the therapeutic procedure. Our institutional review board approved this retrospective observational study.

### Steps of the OCER salvage procedure

#### Endoscopes, devices, and equipment used in OCER

We classified the endoscopes into four groups based on their caliber and size of their working channel relevant to different functions in OCER (Table 1). Groups 1 and 2 comprised LCEs with a 13.2 mm and 10.5–11.7 mm scope tip and a 3.7 and 3.2 mm channel accommodating up to a 10.5F and 9F SEMS delivery system, respectively. From January 2014 to June 2017, the type of colonic SEMS that we used was 22 mm in width with a 10F delivery system (Niti‐S D type colonic stent; TaeWoong Medical Co., Ltd., Goyang‐si, Korea); therefore, Group 1 endoscopes were chosen for both the salvage procedure and standard TTS procedure. However, after a 22 mm width SEMS with a 9F delivery system became available (HANAROSTENT Naturfit; Boston Scientific, Marlborough, MA, USA, or Niti‐S MD type colonic stent; TaeWoong Medical Co., Ltd.), Group 2 endoscopes were chosen more often, and they were exclusively chosen after March 2018. These endoscopes were used alone for the standard TTS procedure in case of accessible TOLs.

**Table 1:** Classification of endoscopes used in over‐the‐catheter endoscope replacement procedure

	Accommodates in channel				Model and size, mm (tip/channel)	Cross‐sectional area^‡^	Original use
	10F DS	9F DS	ERCP catheter^†^	Guidewire			
Large‐caliber endoscope							
Group 1	✔	✔	✔	✔	CF‐H260AI (13.2/3.7)	1.00 (reference)	Standard‐caliber colonoscope with a large channel
Group 2	–	✔	✔	✔	PCF‐H290Z (11.7/3.2)	0.79	Small‐caliber colonoscope
	–	✔	✔	✔	PCF‐Q260JI (10.5/3.2)	0.63	Therapeutic colonoscope
Ultraslim endoscope							
Group 3	–	–	✔	✔	PCF‐PQ260L (9.2/2.8)	0.49	Ultraslim colonoscope
Group 4	–	–	✔	✔	EG‐580NW (5.9/2.4)^§^	0.20	Transnasal gastroscope
			–	✔	EG‐530N (5.9/2.0)^§^	0.20	

^†^Assuming the catheter has a body diameter of 2.3 mm, as in the present study.

^‡^Calculated based on the tip diameter, assigning Group 1 endoscope (CF‐H260AI) as the reference.

^§^The need to exchange the endoscopy systems could have been avoided if endoscopes from the same manufacturer had been available for Group 4, and vice versa.

DS, delivery system; ERCP, endoscopic retrograde cholangiopancreatography.

Groups 3 and 4 comprised ultraslim endoscopes that did not accommodate a SEMS delivery system. These endoscopes were used in the first part of the salvage procedure because of their high insertability. We prioritized the use of the Group 3 endoscope (PCF‐PQ260L; Olympus, Tokyo, Japan), which is an ultraslim colonoscope with a 9.2 mm tip and a 2.8 mm channel that accommodates not only a guidewire (0.035 inch × 450 cm, angle‐tipped, Jagwire; Boston Scientific) but also an ERCP catheter (filiform, 1.6–2.3 mm, tapered, Article No. 0130211; MTW Endoskopie, Wesel, Germany) for fluoroscopic examination of the stricture and better passage of the guidewire through the stricture. However, when the TOL was still inaccessible with the Group 3 endoscope, we selected a Group 4 endoscope to take advantage of its even slimmer shape (5.9 mm tip and 2.0–2.4 mm channel) to access the TOL, although only one Group 4 endoscope model, EG‐580NW (Fujifilm, Tokyo, Japan) had a 2.4 mm channel large enough to pass the ERCP catheter. The whole endoscopy system had to be exchanged when switching to Group 4 endoscopes because of their compatibility with the endoscopy systems, which could have been avoided if endoscopes from the same manufacturer had been available for Group 4. All endoscopic procedures were performed under conscious sedation (intravenous midazolam plus pethidine hydrochloride) using carbon dioxide insufflation in a fluoroscopy‐equipped therapeutic endoscopy unit. All operators who performed the SEMS placement procedures, including OCER, were board‐certified endoscopists with 11–15 years of experience in gastrointestinal endoscopy at the beginning of the study period. The principal interventional endoscopist (Y.S.), who had extensive experience in gastrointestinal stenting, endoscopic submucosal dissection, ERCP, and papillectomy, performed the role of endoscopist or supervisor in most of the SEMS placement procedures (including all OCER procedures) throughout the study period.

#### Performance of OCER

The indication for OCER was the inability to insert an LCE to the TOL, resulting in failure of conventional SEMS placement (Fig. 2A‐1,B‐1). OCER starts with selecting a Group 3 endoscope and accessing and traversing a 0.035 inch angle‐tipped guidewire through the TOL (Fig. 2A‐2,B‐2). Manipulation of an ERCP catheter functions in this process and fluoroscopically delineates the stricture.^12^, ^14^ If the TOL is inaccessible with the Group 3 endoscope, a Group 4 endoscope is selected as a backup for accessing and passing the guidewire through the TOL. If the TOL is accessible with the Group 4 endoscope despite the relatively short length of this scope, the slim scope tip helps to access the stricture orifice without the ERCP catheter (only one of the two Group 4 models used can accommodate the ERCP catheter).

**Figure 3:**
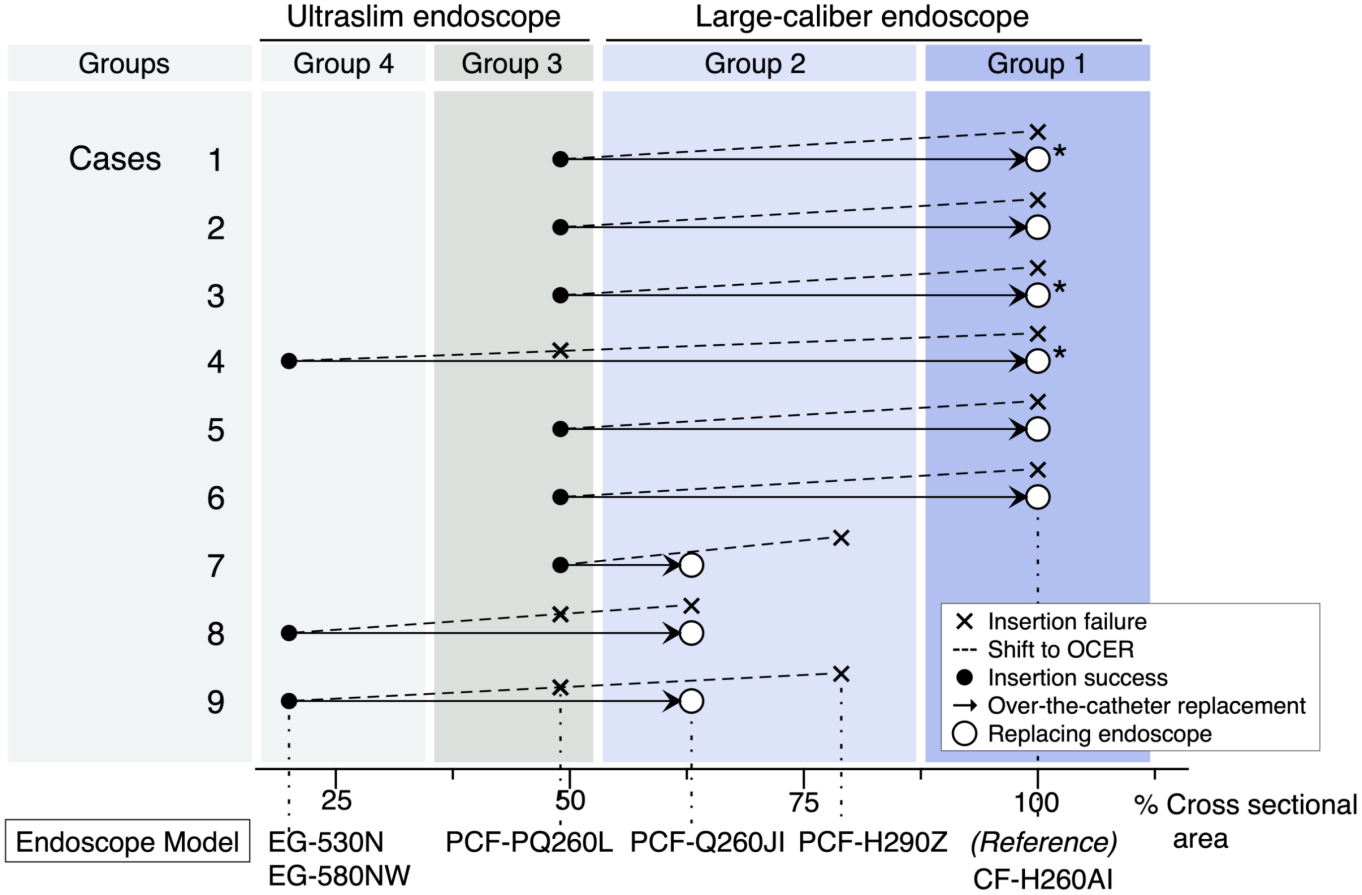
Selection of endoscopes in the course of over‐the‐catheter endoscope replacement (OCER) in nine patients with malignant colonic obstruction. For each patient (cases 1–9), the initially selected large‐caliber endoscope (LCE) that failed to access the target obstructing lesion (TOL) is shown with an X. The endoscope exchange that was intended to initiate OCER is shown with a dotted line. The ultraslim endoscope that was ultimately successfully inserted to the TOL is shown with a black circle, whereas the ultraslim endoscope that failed to be inserted to the TOL is shown with an X. The LCE that was inserted over the catheter is shown with a white circle. These marks correspond to the horizontal axis representing the cross‐sectional area, assigning Group 1 endoscope (CF‐H260AI) as the reference (100%). The endoscope models are identical to those in Table 1. For cases 1, 3, and 4, the replacing LCE could not reach the TOL (denoted by asterisks). Overall, OCER enabled an increase in endoscope sizes to an average of 2.55 times (range, 1.30–5.01) in cross‐sectional area, which was still 2.30 times (range, 1.30–3.17) when limited to the complete replacement cases (*n* = 6). [Colour figure can be viewed at wileyonlinelibrary.com]

The latter part of OCER begins by withdrawing the inserted endoscope and the ERCP catheter, leaving only the guidewire in the colon. Then, outside the body, after inserting the ERCP catheter into the channel of the LCE (Group 1 or 2, depending on the size of the SEMS delivery system) until it protrudes from the scope tip (Fig. 2A‐3a,B‐3a), only the ERCP catheter (not the LCE) is advanced toward the TOL over the guidewire that has been left in place (Fig. 2A‐3b,B‐3b). The aim is to make the catheter traverse the colon and reach the TOL. After the catheter traverses the colon, including its tortuous segment, the next step is insertion of the LCE. The endoscope is advanced using a slow and gentle over‐the‐catheter maneuver combined with the inserted guidewire. Meanwhile, occasionally pulling the catheter helps to keep the catheter‐traversed colon straight and ensure that the advancing endoscope and endoscope‐untraversed colon are oriented in the same direction. This part is completed when the endoscope advances close to the TOL (“complete replacement” of the endoscopes) (Fig. 2A‐4a) or as far as possible (“partial replacement” of the endoscopes) if there is an absolute narrowing of the colon disabling passage of the LCE (Fig. 2B‐4x).

Finally, the last step is removal of the catheter from the endoscopic channel, keeping the guidewire in place, followed by passage of the SEMS delivery system through the stricture (Fig. 2A‐4b,B‐4b) and deployment to the TOL (Fig. 2A‐4c,B‐4c). If the LCE could not be inserted to the TOL (Fig. 2B‐4x), the delivery system itself was advanced for the remaining distance to the TOL over the guidewire under fluoroscopy (Fig. 2A‐4y) to complete the stent placement in a remotely controlled manner (Fig. 2A‐4z,B‐4z).

### Outcomes of OCER in nine patients with inaccessible MCO

All nine patients who underwent OCER were in the palliative setting for malignancies of colorectal (*n* = 4) and extracolorectal (*n* = 5) origins (Table 2). OCER was indicated for these patients because, initially, the LCEs could not access the TOLs of intrinsic (*n* = 3) and extrinsic (*n* = 6) nature at the transverse colon (*n* = 5) and sigmoid colon (*n* = 4) due to computed tomography‐confirmed coexisting peritoneal carcinomatosis (Table 2).

**Table 2:** Clinical characteristics and outcomes of nine patients with malignant colonic obstruction with coexisting peritoneal carcinomatosis who underwent over‐the‐catheter endoscope replacement (OCER)

Case no.	Age (years)/sex	Primary cancer origin	History of abdominal surgery	Site of obstructing lesion	Nature of stricture	Initial LCE insertion	OCER completion	Procedure time (min) OCER/total^†^	Stent diameter × length (mm)	CROSS score^‡^ change	Prognosis (days)
1	71/F	Peritoneum	No	T (S/F)	Extrinsic	RS	Partial (to mid‐S)	29/47	22 × 100	1 → 4	561
2	66/F	Colon (S)	No	S	Intrinsic	Distal S	Complete	43/79	22 × 120	2 → 4	449
3	76/M	Pancreas	No	T (S/F)	Extrinsic	RS	Partial (to SDJ)	56/108	22 × 120 22 × 120	1 → 4	51
4	55/F	Ovary	Yes	S	Extrinsic	RS	Partial (to mid‐S)	89/155	22 × 120 22 × 100 22 × 80	0 → 3	36
5	49/F	Stomach	No	T	Extrinsic	Mid‐S	Complete	37/64	22 × 120	0 → 3	182
6	75/M	Rectum, resected	Yes	S	Extrinsic	RS	Complete	34/83	22 × 100 22 × 120	0 → 4	136
7	69/M	Bladder	Yes	T (S/F)	Extrinsic	Distal S	Complete	39/77	22 × 120	0 → 2	32
8	54/M	Colon (S)	No	S	Intrinsic	RS	Complete	60/108	22 × 90	0 → 1	69
9	87/F	Colon (T)	No	T	Intrinsic	Distal S	Complete	81/111	22 × 120	0 → 4	Unknown

^†^ For OCER: from ultraslim endoscope insertion to completion of self‐expandable metallic stent (SEMS) deployment, including two sequential ultraslim endoscope insertions requiring endoscope system exchanges in cases 4, 8, and 9. For Total: from initiation of anesthesia to end of the procedure, including multiple SEMS placements in cases 3, 4, and 6.

^‡^ CROSS score^12^, ^13^ represents levels of oral intake on a five‐grade scale: 0, continuous decompression required; 1, no oral intake; 2, liquid or enteral nutrient intake; 3, soft solid oral intake with symptoms of stricture; 4, soft solid oral intake without symptoms of stricture.

CROSS, ColoRectal Obstruction Scoring System; F, female; LCE, large‐caliber endoscope; M, male; RS, rectosigmoid; S, sigmoid colon; S/F, splenic flexure; SDJ, sigmoid descending junction; T, transverse colon.

In the first step of OCER, ultraslim Group 3 endoscopes (*n* = 9) were selected. Insertion to the TOL failed in three of these cases. However, after Group 4 endoscopes were chosen for these three patients, insertion to the TOL was successful, ultimately resulting in access to the TOL in all nine patients (Fig. S1). This was followed by successful passing of a guidewire through the strictures, although no scope could pass the stricture. As the next step, complete (*n* = 6) or partial (*n* = 3) replacement (Fig. 3) of the endoscopes by LCEs was achieved in all nine patients, which led to successful SEMS placement at the TOL that caused MCO using 10F (for cases 1–6) or 9F (for cases 7–9) SEMS delivery systems (Fig. 3). Notably, this was conducted remotely in three patients with partial replacement (cases 1, 3, and 4). Two to three stents per procedure were needed because of long lesions in two cases (cases 3 and 4) and to avoid the distal stent edge off from vertically contacting the colonic wall in one case (case 6) (Table 2). The duration of the procedure (mean ± standard deviation) was 38.2 ± 16.5 min for failed standard procedures (from induction with sedatives through initial LCE insertion to the beginning of OCER), 52.0 ± 21.3 min for net OCER (from ultraslim endoscope insertion to the end of the procedure), and 90.2 ± 33.7 min for the total procedure (from induction with sedatives to the end of the procedure) (Table 2). More specifically, the mean time from insertion of an ultraslim endoscope that reached the TOL (Group 4 in cases 4, 8, and 9) to the guidewire passage was 11.9 ± 7.5 min, and that from the reinsertion of the LCE in an over‐the‐catheter manner to stent deployment (first stent in cases 3, 4, and 6) was 18.4 ± 5.2 min. A series of representative images of OCER in case 1 is shown in Figure S2. No procedure‐related adverse events occurred, and the patients' symptoms improved in the short term. The prognosis was variable depending on the underlying malignant diseases, with a median survival time of 102.5 days (range, 32–561 days) (Table 2). OCER enabled an endoscope size increase in cross‐sectional areas to an average of 2.55 times (range, 1.30–5.01), which was still 2.30 times (range, 1.30–3.17) when limited to complete replacement (*n* = 6) (Fig. 3).

## DISCUSSION

We have herein reported for the first time that the salvage procedure OCER is applicable for SEMS placement when the TOL is inaccessible with an LCE in patients with MCO with coexisting peritoneal carcinomatosis. OCER had a high success rate of preventing impending technical failure of stenting in nine patients.

There are two critical procedural steps in OCER: the first is accessing the TOL through the tortuous colon, and the second is replacing the endoscopes over the catheter. For the first step, a Group 3 endoscope (PCF‐PQ260L; Olympus), which has high insertability^9^, ^10^ and a small caliber (50% smaller cross‐sectional area than a Group 1 endoscope) (Fig. 3), was successfully inserted in six of nine (66.7%) patients. Moreover, for the three highly challenging cases in which a Group 3 endoscope failed, the smallest‐caliber Group 4 endoscope functioned as the backup, leading to a successful first step in all nine cases (Fig. 3, Fig. S2).

For the second step, the unique use of the ERCP catheter plays a critical role. It is extremely important to advance solely the catheter first, which very smoothly follows the guidewire. This smooth advancement is facilitated by its 1.6 mm tip that gradually tapers to a 2.3 mm body, with the distal flexible part of 28 cm having no metal stylet within the lumen. Once the catheter has been inserted along the guidewire, the catheter body has adequate rigidity because of the metal stylet contained within, over which the LCE is efficiently navigated. We speculate that the catheter keeps the colonic tortuous segment straight and linear to the advancing endoscope, avoiding angulation, friction, and loss of force transmission. In six of nine patients, the LCE reached the TOL (complete replacement), overcoming the relative luminal narrowing by invasion and the abnormal tissue fixation by adhesion that had initially hindered its advancement. In three patients, however, the LCE became stuck in a region of absolute narrowing, leaving a distance to the TOL (partial replacement). However, SEMS placement succeeded in a remotely controlled manner (Figs 2A‐4z, B‐4z,3). The rationale is that the delivery system gains distance in the endoscope channel and can travel the remaining distance over the wire. This remotely controlled technique adds to the significance of OCER as a salvage procedure.

There might be large anatomical variations in colons affected by peritoneal carcinomatosis^6^ that could pose concerns in terms of safety or technical adequacy when conducting OCER. However, the abovementioned procedural steps in OCER reasonably alleviate possible safety concerns related to its use: switching to an ultraslim endoscope^9^, ^10^, ^11^ appears to be safer when investigating the colon with irregular structural alterations. The reinsertion of an LCE using the over‐the‐catheter technique might lessen the friction against the colon. Furthermore, reinsertion is exempted from the absolute necessity to cross the entire colonic segment involved in the peritoneal carcinomatosis. Potential technical failures unique to OCER include failure to insert even an ultraslim endoscope or to advance the delivery system to the TOL during the remotely controlled technique, although these scenarios, at least partially, might be related to the presence of multiple TOLs; therefore, the occasional placement of an additional SEMS might be considered.

One forthcoming trend in SEMS delivery systems is the reduction of their diameter. Recently, 9F SEMS delivery systems that qualify a relatively small‐caliber endoscope (Group 2 endoscope) have become available.^15^ However, there are still challenging cases of MCO for which a standard TTS procedure fails. Moreover, 10F delivery systems are still used worldwide. Thus, we expect that a substantial number of patients with MCO who have peritoneal carcinomatosis with colon involvement require a salvage procedure for challenging SEMS placement because MCO of extrinsic or extra colorectal etiology is associated with unsuccessful stenting.^6^, ^7^, ^14^, ^16^, ^17^, ^18^

Our herein‐described new salvage procedure is unique in maximizing the functions of different caliber endoscopes in a complementary way while requiring only endoscopes and devices already widely available. Thus, the introduction of OCER would be more feasible for most institutions routinely practicing endoscopic stenting than, for instance, switching to a radiological “over‐the‐wire” procedure for SEMS placement, which requires distinct expertise and has not been confirmed to be helpful in patients who have MCO with peritoneal carcinomatosis. Indeed, OCER might be applicable to various clinical situations in which an LCE is favored but difficult to insert to the target.

There were some limitations in the present study. First, OCER has evolved from a single‐center experience, which might have resulted in a biased selection of those indicated for endoscopic stenting among the whole MCO patient cohort. However, the procedures for consecutive patients sent for stenting were not declined by our endoscopists unless there were absolute contraindications; therefore, patients who underwent OCER are thought to represent a group of patients encountered with some frequency when managing MCO. Second, the number of OCER cases included in this analysis was small; indeed, only nine patients in our study cohort were indicated for OCER because of TOL inaccessibility. However, these patients accounted for the largest portion of the impending technical failures (9 out of 12, Fig. 1) in palliative settings in our study cohort, and this highlights the importance of TOL accessibility when stenting and the significance of the salvage procedure. A prospective, large‐scale, multicenter study is required to clarify these points.

In conclusion, OCER is a potentially feasible and valuable option for salvage of SEMS placement in patients with coexisting MCO and peritoneal carcinomatosis. Improvement in the technical success rate of SEMS placement by OCER has important implications, especially for the palliative care of patients who are poor surgical candidates.

## CONFLICT OF INTEREST

AUTHOR E.I. has received lecture honoraria from Takeda Pharmaceutical Co., Ltd and belongs to an endowed course supported by the following companies: Ono Pharmaceutical Co., Ltd, Miyarisan Pharmaceutical Co. Ltd, Sanwa Kagaku Kenkyusho Co., Ltd, Otsuka Pharmaceutical Factory, Inc., Fujifilm Medical Co., Ltd, Terumo Corporation, Fancl International, Inc., and Ohga Pharmacy. The other authors declare no conflict of interest for this article.

## FUNDING INFORMATION

None.

## Supporting information


**Figure S1** Flowchart of endoscope selection in nine patients who underwent the salvage procedure (over‐the‐catheter endoscope replacement [OCER]). First, ultraslim Group 3 endoscopes (*n* = 9) were selected. Group 4 endoscopes were selected after the failed insertion of Group 3 endoscopes to the target obstructing lesion (TOL). Ultimately, access to the TOL was successful in all nine patients, which was followed by the successful passage of a guidewire (GW) through the strictures and successful self‐expandable metallic stent (SEMS) placement. ERCP, endoscopic retrograde cholangiopancreatography; LCE, large‐caliber endoscope.

**Figure S2** A representative patient (case 1) with malignant colonic obstruction (MCO) with coexisting peritoneal carcinomatosis undergoing self‐expandable metallic stent (SEMS) placement using over‐the‐catheter endoscope replacement (OCER). (A) The MCO associated with peritoneal carcinomatosis was observed at the splenic flexure on computed tomography (yellow arrowhead). Massive ascites and coexisting intrapelvic carcinomatosis were seen (red arrowhead). (B, C) Representative images showing SEMS placement using OCER. An ultraslim endoscope (Group 3) was inserted to the target obstructing lesion (B, yellow arrowhead), followed by an endoscopic retrograde cholangiopancreatography catheter combined with a guidewire (B, red arrowhead) passed through the stricture. Although the replaced large‐caliber endoscope could not be advanced past the sigmoid colon by OCER (C, yellow arrowhead), SEMS placement using a 10F delivery system was completed in a remotely controlled manner (C, red arrowhead).

**Table S1** Clinical characteristics of the 165 patients who underwent endoscopic stenting during the study period.
