# Supplementary figures and images for: Over‐the‐catheter endoscope replacement for stenting in patients with inaccessible malignant colonic obstruction with coexisting peritoneal carcinomatosis
Source: Dig Endosc. 2022 Aug 9;34(7):1481–90. doi: 10.1111/den.14385 (PMC9804792; doi:10.1111/den.14385)

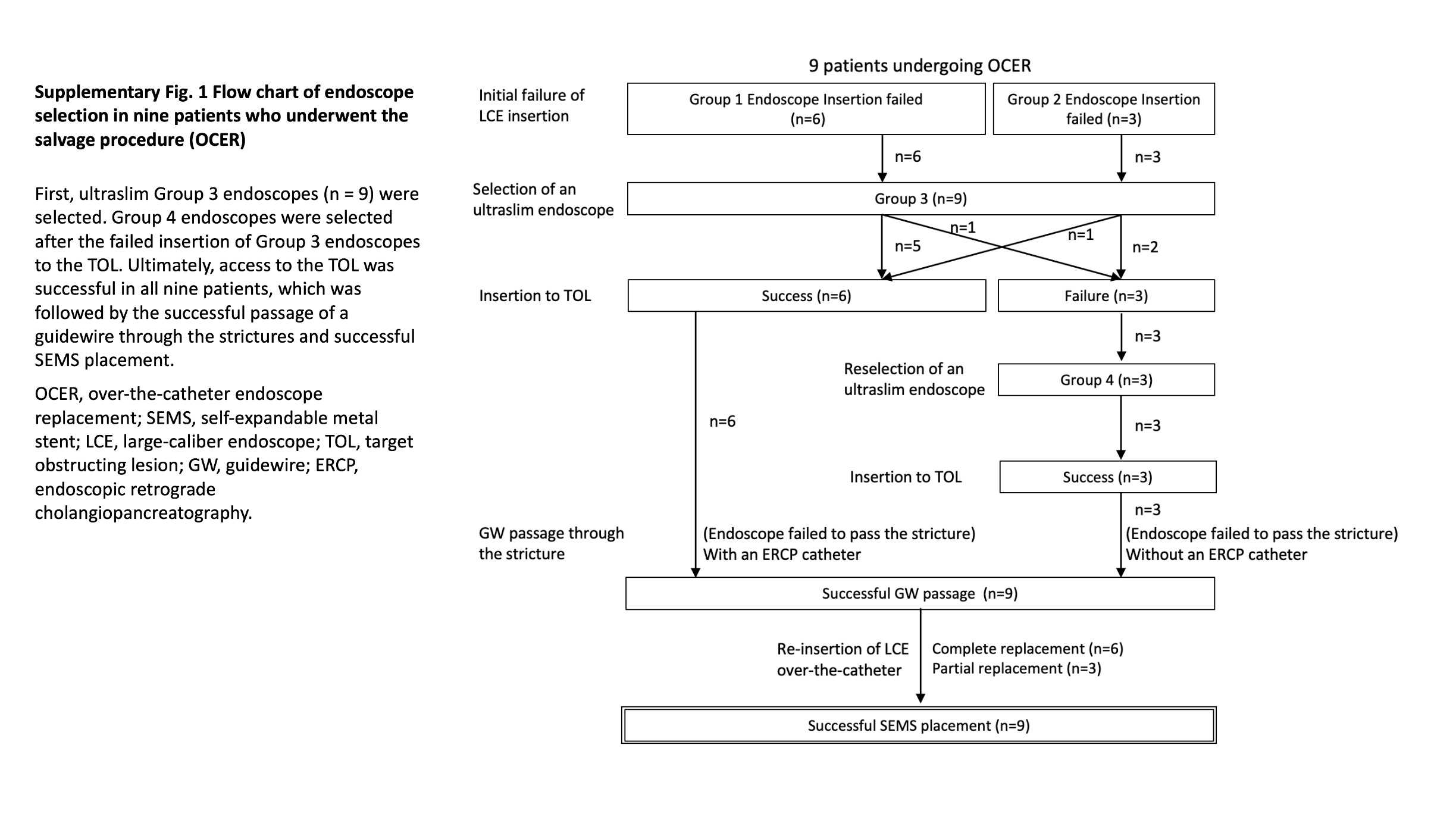

Supplement: Supplementary file 1 — Figure S1 Flowchart of endoscope selection in nine patients who underwent the salvage procedure (over‐the‐catheter endoscope replacement [OCER]). First, ultraslim Group 3 endoscopes (n = 9) were selected. Group 4 endoscopes were selected after the failed insertion of Group 3 endoscopes to the target obstructing lesion (TOL). Ultimately, access to the TOL was successful in all nine patients, which was followed by the successful passage of a guidewire (GW) through the strictures and successful self‐expandable metallic stent (SEMS) placement. ERCP, endoscopic retrograde cholangiopancreatography; LCE, large‐caliber endoscope. [file DEN-34-1481-s001.tiff]

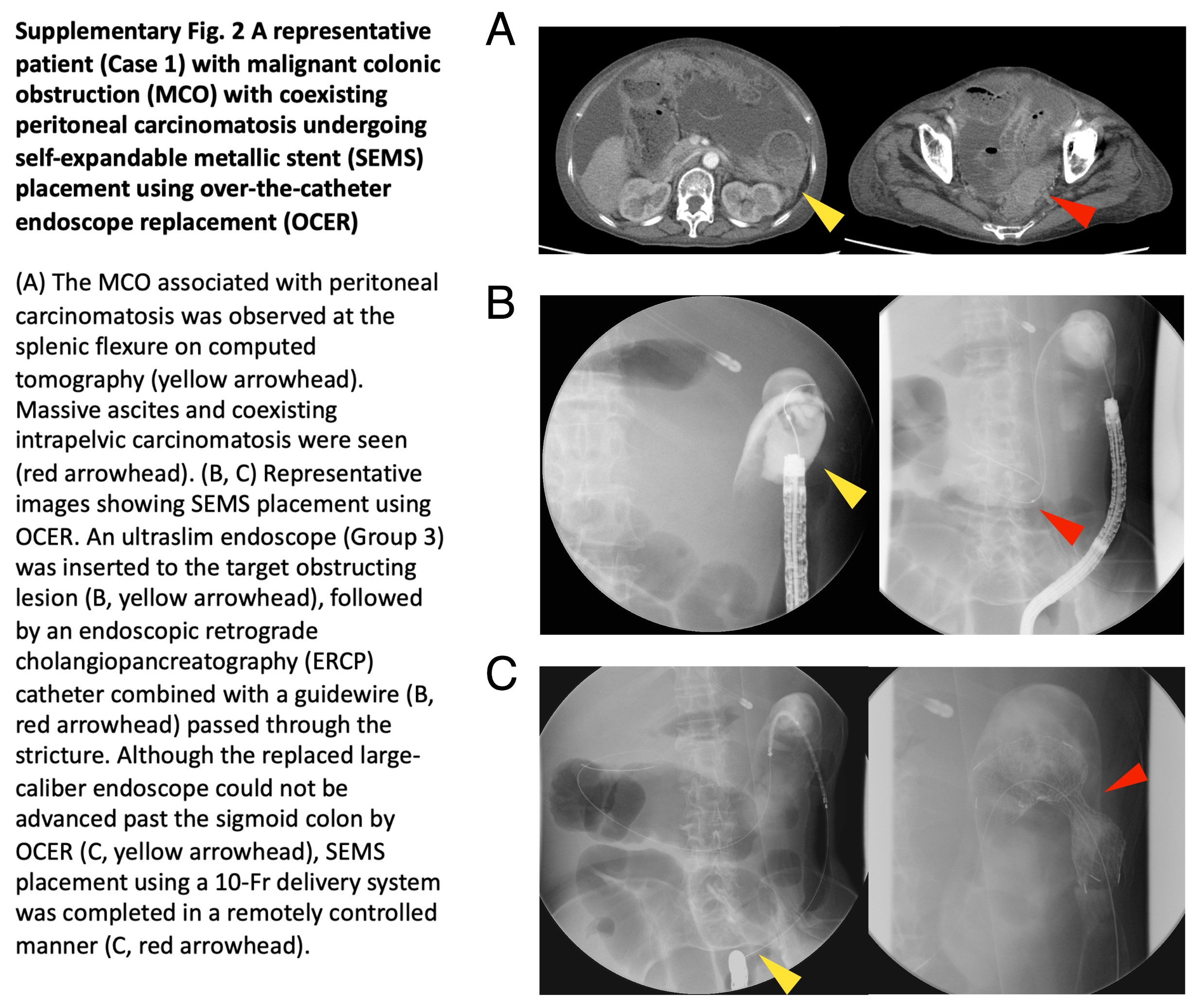

Supplement: Supplementary file 2 — Figure S2 A representative patient (case 1) with malignant colonic obstruction (MCO) with coexisting peritoneal carcinomatosis undergoing self‐expandable metallic stent (SEMS) placement using over‐the‐catheter endoscope replacement (OCER). (A) The MCO associated with peritoneal carcinomatosis was observed at the splenic flexure on computed tomography (yellow arrowhead). Massive ascites and coexisting intrapelvic carcinomatosis were seen (red arrowhead). (B, C) Representative images showing SEMS placement using OCER. An ultraslim endoscope (Group 3) was inserted to the target obstructing lesion (B, yellow arrowhead), followed by an endoscopic retrograde cholangiopancreatography catheter combined with a guidewire (B, red arrowhead) passed through the stricture. Although the replaced large‐caliber endoscope could not be advanced past the sigmoid colon by OCER (C, yellow arrowhead), SEMS placement using a 10F delivery system was completed in a remotely controlled manner (C, red arrowhead). [file DEN-34-1481-s003.tiff]
