# Supplementary material for: Over‐the‐catheter endoscope replacement for stenting in patients with inaccessible malignant colonic obstruction with coexisting peritoneal carcinomatosis
Source: Dig Endosc. 2022 Aug 9;34(7):1481–90. doi: 10.1111/den.14385 (PMC9804792; doi:10.1111/den.14385)
Supplement: Supplementary file 3 — Table S1 Clinical characteristics of the 165 patients who underwent endoscopic stenting during the study period. [file DEN-34-1481-s002.docx]

Supplementary Table 1 Clinical characteristics of the 165 patients who underwent endoscopic stenting during the study period.

|  | Bridge to surgery (n = 87) | Palliative (n = 78) |
| --- | --- | --- |
| Sex, M/F, % | 55.2/44.8 | 55.1/44.9 |
| Age, years | 70.4 ± 10.4 | 67.5 ± 14.2 |
| Location of obstruction |  |  |
| Ascending colon* | 7 (8.0) | 11 (14.1) |
| Transverse colon† | 13 (14.9) | 14 (17.9) |
| Descending colon | 8 (9.2) | 7 (9.0) |
| Sigmoid colon | 41 (47.1) | 24 (30.8) |
| Rectum | 18 (20.7) | 22 (28.2) |
| Nature of obstruction |  |  |
| Intrinsic/extrinsic, % | 98.9/1.1 | 60.3/39.7 |
| Origin of malignancy |  |  |
| Colorectum | 86 (98.9) | 52 (66.7) |
| Stomach |  | 13 (16.7) |
| Biliary/pancreas |  | 5 (6.4) |
| Genitourinary tract |  | 4 (5.1) |
| Peritoneum/unknown |  | 2 (2.6) |
| Breast |  | 1 (1.3) |
| Lung |  | 1 (1.3) |
| Other (inflammatory) | 1 (1.1) |  |

Data are presented as mean ± standard deviation or n (%) unless otherwise noted.

M, male; F, female

*Including ileocecal segment

†Including hepatic and splenic flexures
